# Quantitative spectral assessment of intracardiac electrogram characteristics associated with post infarct fibrosis and ventricular tachycardia

**DOI:** 10.1371/journal.pone.0204997

**Published:** 2018-10-05

**Authors:** John Morellato, William Chik, M. A. Barry, Juntang Lu, Aravinda Thiagalingam, Pramesh Kovoor, Jim Pouliopoulos

**Affiliations:** 1 University of Sydney, Sydney, Australia; 2 Department of Cardiology, Westmead Hospital, Sydney, Australia; University of Minnesota, UNITED STATES

## Abstract

**Background:**

Post-myocardial infarction (MI) remodeling contributes to increased electrophysiological and structural heterogeneity and arrhythmogenesis. Utilising the post-infarct ovine model our aim was to determine unipolar electrogram frequency characteristics consequent to this remodeling and the development of Ventricular Tachycardia (VT).

**Methods and results:**

Mapping studies were performed on 14 sheep at >1 month post-MI induction. Sheep were divided into VT inducible (n = 7) and non-inducible (n = 7) groups. Multielectrode needles (n = 20) were deployed within and surrounding ventricular scar for electrophysiological assessment of electrogram amplitude and width. Spectral analysis of electrograms was undertaken using wavelet and fast fourier transformations (WFFT) to calculate root mean square (RMS) power intervals spanning 0-300Hz in 20Hz intervals. Quantitative assessment between electrophysiological and histological parameters including collagen density, and structural organization of the myocardium was performed.

Increasing myocardial scar density resulted in attenuation of electrogram amplitude and RMS values. (all *p*<0.01). Between groups there were no differences in electrogram amplitude (*p* = 0.37), however WFFT analysis revealed significantly higher RMS values in the VT group (*p*<0.05) in association with high frequency fractional components of the electrogram. As scar density increased, greater between-group differences in RMS were observed spanning this high frequency (200-280Hz) spectrum and which were proportionally dependent on the degree of structural disorganisation of the myocardium (*p<*0.001) and number of extrastimuli required to induce VT (*p*<0.05).

**Conclusion:**

High frequency unipolar electrogram spectral characteristics were quantitatively co-influenced by the presence of fibrosis and degree of myocardial structural dissorganisation and were associated with the propensity for development of VT.

## Introduction

Catheter based mapping and ablation is an important therapeutic option in patients with post-myocardial infarct (MI) ventricular tachycardia (VT).[1] Despite the advances in technology including irrigated radiofrequency ablation, 3-dimensional mapping and ablation, ablation efficacy can be influenced adversely by the complexity of the scar substrate and by the location of reentrant circuits which may be concealed deep within the mid-myocardial layers.[2–4]

The structural remodeling that occurs after myocardial infarction contributes to increased electrophysiological heterogeneity attributing to electrogram fractionation which is a result of discontinuous propagation through myocardial bundles that are separated by fibrous tissue.[5–9] Electrogram fractionation, conventionally recorded using bipolar catheter modalities, has been a feature in a number of post-myocardial infarction VT studies.[2, 5, 10–12] With increasing utilization of unipolar mapping modalities in clinical electroanatomical mapping, experimental evidence suggests that unipolar recordings are equally as sensitive as bipolar recordings in detecting fractionation.[13, 14] Despite suggestions that electrogram fractionation indicated within areas of myocardial scarring may be useful for targeting VT ablation therapy, the utility of this marker for identification of critical arrhythmogenic substrate associated with VT has previously not been investigated quantitatively with respect to myocardial structure.[6, 10, 11]

Since bipolar recordings are inherently dependent on the directional nature of the bipole orientation with respect to the angle of the propagating wavefront, the non-directional property of the unipolar recording may provide a benefit in analyzing wavefront characteristics in relation to the discontinuity of propagation associated with myocardial remodeling.[14]

Robust detection techniques have allowed us to refine our analysis of the electrogram. Fast Fourier Transform and wavelet analysis techniques have been described for analysis of microvolt amplitude, high frequency potentials that occur within the QRS complex of fractionated electrograms. [15–17] Mathematical models predict that discontinuous activation in heterogeneous myocardium gives rise to high frequency perturbations that are detectable in the unipolar electrogram frequency spectrum between 100–300 Hz. [18]

Utilising the ovine model of post-infarct cardiomyopathy, the purpose of this study was to quantitatively investigate, how unipolar electrogram frequency characteristics are influenced by electrophysiological and structural heterogeneity post-MI and whether changes in these characteristics are associated with the propensity for reentrant ventricular tachycardia.

## Methods

### Preparation of animals used for the study

The study was approved by the Sydney West Animal Research Ethics Committee and conformed to the guidelines set for animal research by the National Health and Medical Research Council, Australia.

Experiments were performed on 14 castrated male sheep (57 ± 15kg). The sheep were sedated with intramuscular xylazine (0.5mg/kg) and anaesthesia was induced with an intravenous bolus of propofol (4mg/kg) prior to intubation. General anaesthesia (GA) was maintained with 1–4% isoflurane in 100% oxygen and intravenous NaCl (100mL/hr) was maintained throughout the procedure. Anterior MIs were induced in all sheep by inflating an angioplasty balloon thereby totally occluding the ovine left anterior descending artery beyond the first diagonal branch or its equivalent for 3 hours as described by Reek *et al*. [19] Sotalol was administered at an oral dose of 40 mg twice daily post-MI to reduce the risk of fatal arrhythmias. Sotalol was discontinued for over 5 days (mean of 11 half-lives) prior to the electrophysiology study.

An electrophysiology study was performed 12 ± 16 weeks post-MI to determine whether VT was inducible using standardised protocols. General anaesthesia was induced and maintained as described for the MI induction procedure. A standardized protocol for induction of VT was attempted using programmed ventricular stimulation from the right ventricular apex, with a drive train of 8 paced beats at a cycle length of 400ms and up to a maximum of 4 extrastimuli at twice the capture threshold.[20–23] Each extrastimulus was decremented at 10ms interval until refractoriness. If VT was not inducible after 2 attempts from the right ventricular (RV) apex, the same induction protocol was repeated from the RV outflow tract. Sheep with inducible monomorphic VT lasting >10 seconds were designated as VT inducible (VT_+_) with the remaining sheep allocated to the VT-non inducible (VT-) group. Sheep with inducible VT were again administered sotalol at a dose of 40 mg twice-daily post-procedure which was discontinued 5-days prior to the mapping procedure.

### Mapping procedure

Detailed ventricular mapping and repeat electrophysiology studies were performed on all sheep at >1-month after the index electrophysiology study. Longitudinal assessment in this regard was performed to confirm temporal consistency in electrophysiology study outcomes. While the sheep was maintained under GA, a left thoracotomy was performed through the 4th intercostal space to expose the heart. In all sheep, a minimum of 20 multi-electrode plunge needles were deployed via the epicardium. The intramural needles spanned from the apex to the base, covering the anterolateral and posterolateral regions of the left ventricle (LV) in a grid array configuration. From the epicardial aspect, the needles were strategically positioned in one of four types of tissues: i) scarred myocardium, ii) at the periphery of scarred myocardium, iii) in normal myocardium adjacent to the area of scar (iv) in normal myocardium distant from the scar zone. The location of scar tissue during needle insertion was recorded by visual inspection referenced to anatomical landmarks and palpation. The needle length varied dependent on electrode configuration which ranged from 2 to 4 electrodes (electrode length = 1.5mm, interelectrode distance = 1.5mm, and external diameter = 0.8mm). The designated needle type (2–4 electrode) during deployment was determined by estimation of myocardial wall thickness upon palpation. A minimum of 60 electrodes (5 x 2-pole, 10 x 3-pole, and 5 x 4-pole needles) were deployed in each heart.

The needle electrodes were configured for unipolar recording with a chest wall retractor connected as the unipolar reference.

### Data collection

After a 30-minute settling period, unipolar electrograms from each plunge needle electrode were simultaneously sampled on the Prucka Cardiolab system (GE Healthcare, USA) during sinus rhythm at 1 kHz, and filtered at 0.05 – 500Hz. The electrograms were exported into in-house customised software utilising the Matlab programming language for offline analysis.

At the completion of the study, the sheep were euthanized and numbered markers were sutured onto the epicardial surface to annotate individual intramural needle locations. The heart was excised and bathed in 10% formalin solution for 2 weeks in preparation for histological analysis of the myocardium.

### Histological analysis

After fixing the entire heart specimen in formalin, a transmural block of myocardium (1cm x 1cm x transmural thickness cm) surrounding each needle was excised. The samples ware then dehydrated with 100% ethanol and embedded in paraffin wax. A 4-μm-thick section along the needle tract was cut from each block and stained with Gomori trichrome.

**Fig 1:**
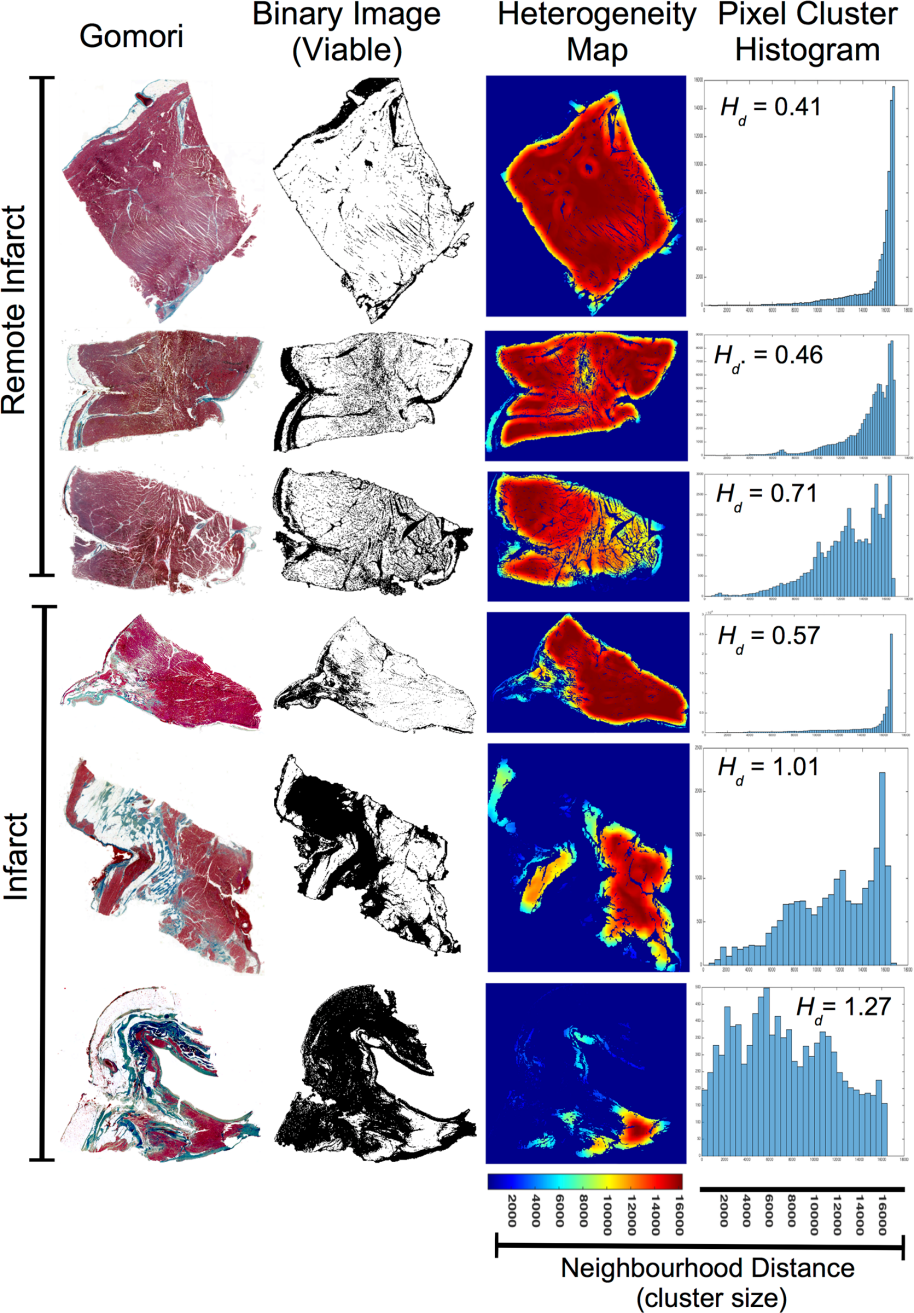
Histological analysis of Gomori trichrome stained myocardial sections from infarcted and non-infarcted sites demonstrating variation in heterogeneous distribution of viable myocardium; *Gomori*: Blue signifying collagen and red signifying viable myocardium. ***Binary Image***: Automated discrimination of viable myocardium (white), from non-viable myocardium (black), using digital image processing software. ***Heterogeneity Map***: Map of *H*_*i*_ (see Eq 1 in methods), where colour blue to red indicates increasing neighbourhood distance (clustering) of viable tissue or collagen specific pixels (only viable tissue shown as example). ***Pixel Cluster Histogram***: Histogram of *H*_*i*_ (where y axis = number of pixels, and x axis = cluster distance). ***H*_*d*_**: Heterogeneity index, where small value indicates multiple small clusters of tissue separated by greater distances, and large values indicate a small number of large clusters separated by small distances or single clusters. All tissue sections have equal scale.

The method for automated histological analysis of collagen deposition in myocardium has been previously described as follows.[24] Each tissue section was digitally scanned at 4000 pixels per inch and imported into in-house histology analysis software. The software was able to differentiate between viable myocardium and collagen in scar, based on a threshold algorithm utilising the red and blue colouring of pixels (Fig 1). The total area in pixels of the myocardial section and epicardial-endocardial tissue thickness was determined using imaging software (ImageJ). The quantity of viable myocardium and collagen was calculated as the percentage of red or blue color-stained pixels respectively, expressed as per unit area of the myocardial section. The amount of scar within each tissue slice was reported as the *(total tissue area in pixels–the total area of viable myocardium in pixels) ÷ 100*.

Each digitised tissue section was then imported into Matlab (r2014b, The Mathworks Inc. MA, USA) for offline conversion into separate binary images (0,1) based on thresholding for viable myocardium (Fig 1) and collagen respectively. The heterogeneous distribution (*H*_*d*_*)* of viable myocardium within each tissue section was calculated using the following equations:

$$\begin{array}{l} H_{i}=\left(\begin{array}{c} n\\ \vdots \\ i=1 \end{array}\right)\frac{\sum_{j=1}^{n}\sqrt{{\left(px_{i}-px_{j}\right)}^{2}+{\left(py_{i}-py_{j}\right)}^{2}}}{n} \end{array}$$ (1)

$$H_{d}=\frac{{}^{P95}H_{i}{-}^{P5}H_{i}}{{}^{P50}H_{i}}$$ (2)

Where *p* = pixel at coordinates *x*,*y*, of the image region identified using pixel colour thresholding (red = viable); *n* = the total number of pixels adhering to criteria for *p*; *H*_*i*_ = vector of average neighbourhood distances for each *p*; *P* = percentile (within distribution of *H*_*i*_); *H*_*d*_ = Heterogeneity index, where *H*_*d*_ = 0 infers clustering around a centre point, *H*_*d*_ > 0 infers increased dispersion of clusters.

### Analysis of visual electrogram characteristics

Electrodes with inadequate myocardial contact as determined by manual offline histological assessment were excluded from analysis.

**Fig 2:**
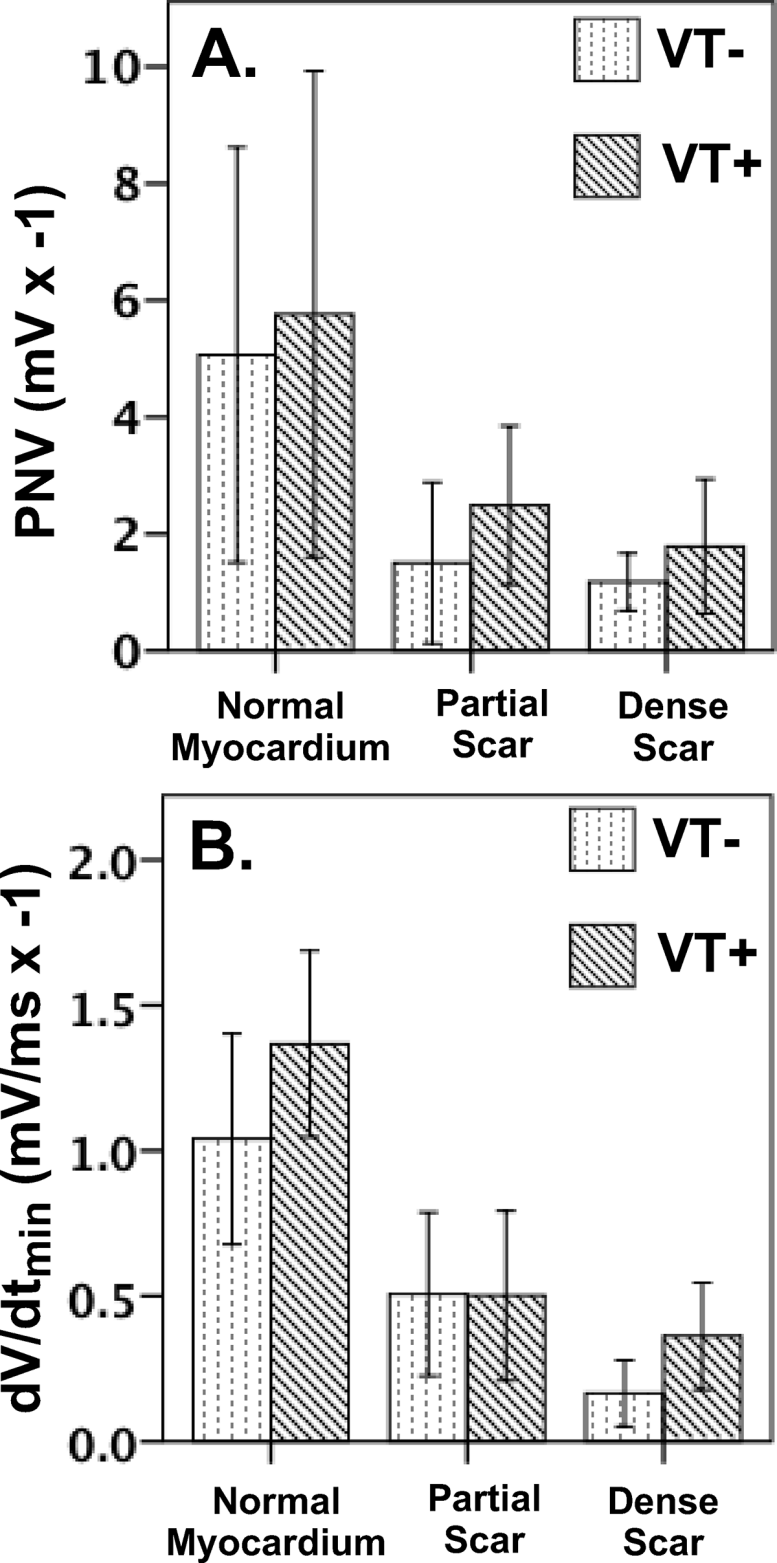
***A***: Effect of myocardial pathology on unipolar electrogram peak negative voltage (PNV). There was no significant difference between the degree of scarring and electrogram amplitude between groups (all *p*>0.05). ***B***: Effect of myocardial pathology on unipolar electrogram dV/dT_min_. Similarly, there was no significant difference in the relationship between the degree of scarring and electrogram dV/dt_min_ between groups (all *p*>0.05).

Visual electrogram characteristics were defined based on criteria relating to electrogram amplitude, and local rate of depolarization (Fig 2). The QRS window for all local electrograms was selected to span the time from the earliest intrinsic deflection of the QRS to 30ms after the terminal portion of the surface ECG R-wave. The rate of unipolar electrogram depolarization which is indicative of activation of sodium mediated currents, was defined as the dV/dt_min_ of the QRS complex averaged over a 4 ms sliding window.[25] Electrogram amplitude, reported as peak negative voltage (PNV) was calculated as the QRS amplitude below the iso-voltage mark.

### Frequency spectrum analysis—(i) Discrete Wavelet Transform (DWT)

**Fig 3:**
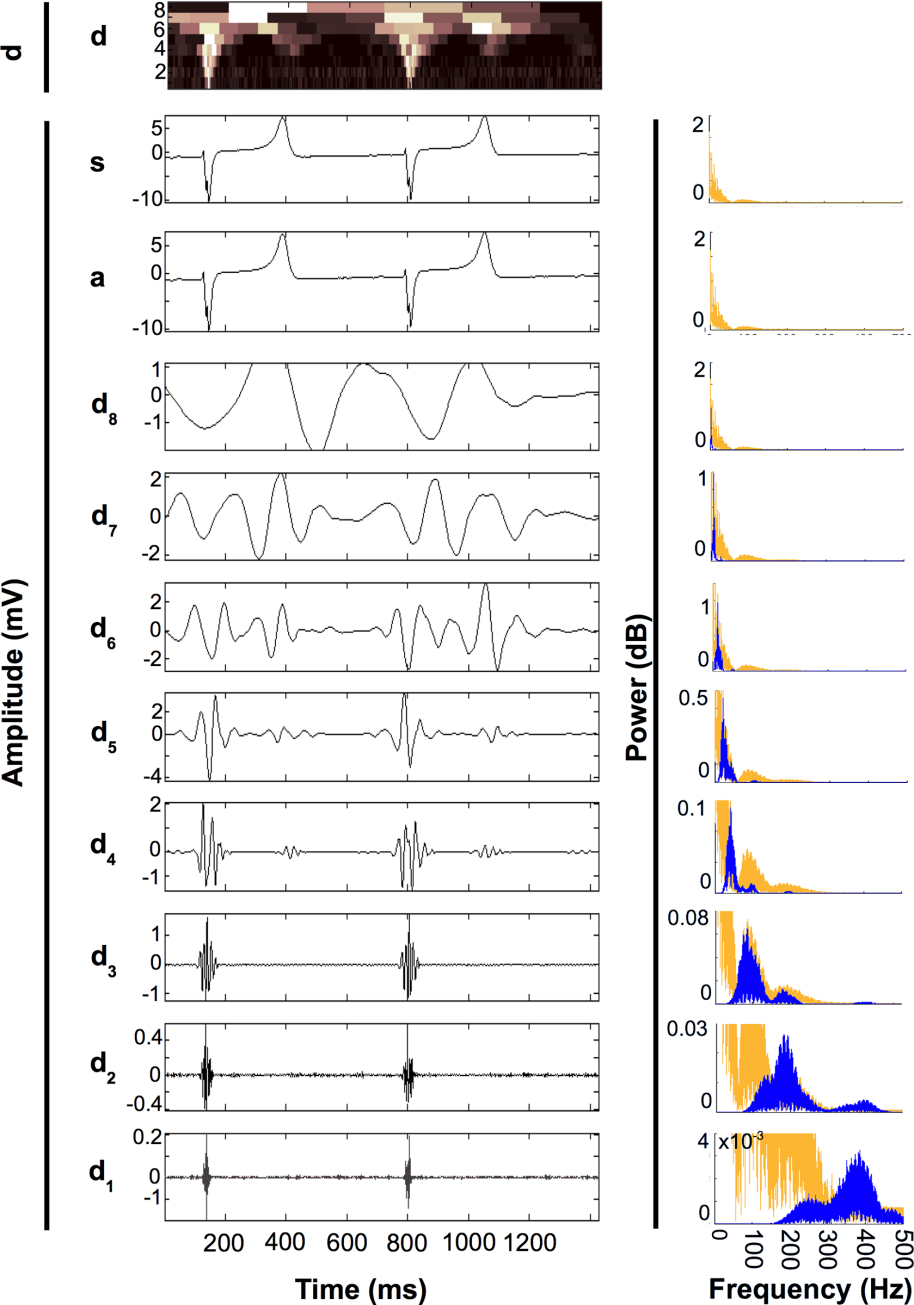
Example of 8 level decomposition (d_1_ –d_8_) and approximation (a) of a unipolar electrogram signal (s). **d**. (***top***) Superimposed plot of electrogram details for all decomposition levels. Panels (***right***) show spectral frequency distributions using fast fourier transformation (FFT) of s (orange) and corresponding decompositions (blue). Microvolt amplitude features extracted at low decomposition levels (e.g d_1_ –d_2_), demonstrate high frequency content, and represent characteristics of the QRS complex. Millivolt amplitude features extracted at higher decomposition levels, (d_4_ –d_7_) reflect higher power but lower frequency characteristics representative of the QRS and T-wave complex. Summation of decomposed signals d_1_ to d_8_ equates to the approximation (a) which is identical to the original signal (s).

**Fig 4:**
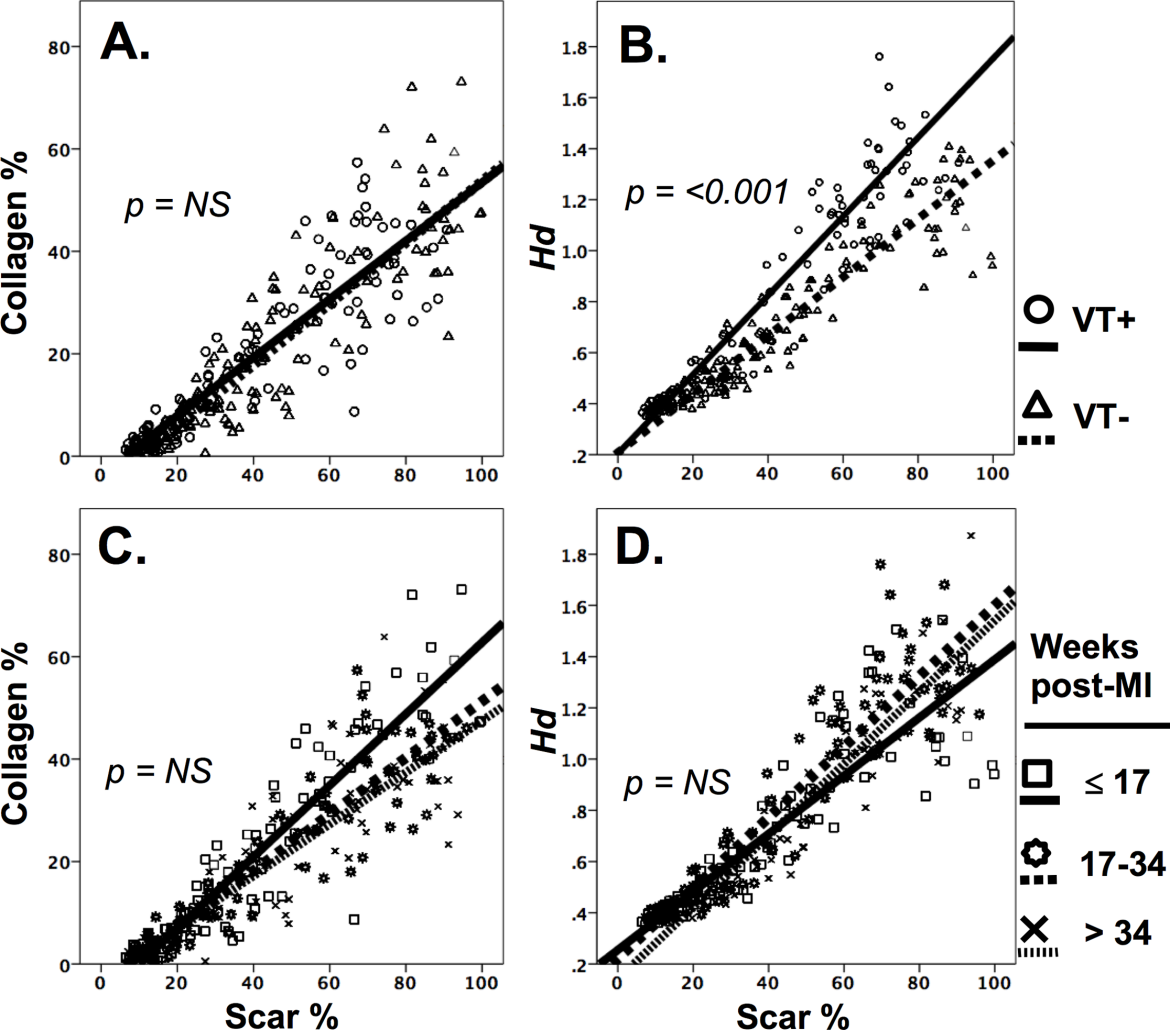
Structural variation of myocardium between groups. **A**. Relationship between total scar density and collagen density between groups. This relationship is similar between VT+ and VT- groups. **B**. Relationship between scar density and heterogeneity index (*H*_*d*_) between VT+ and VT- groups. As scar density increases, the structure of viable myocardium becomes increasingly heterogeneous to a greater degree in the VT+ group compared to the VT- group. **C**. Relationship between scar density and collagen density with respect to infarct maturity. **D**. Relationship between scar density and *H*_*d*_ with respect to infarct maturity. Non-significant trends demonstrating reduced collagen density:scar density ratio with increasing infarct maturity, and an increased *Hd*:scar density ratio with increasing infarct maturity.

Multiresolution electrogram analysis was conducted using the DWT (Fig 3) to describe the content and range of frequencies, specific to morphological features of electrograms recorded from different sites within the left ventricle, that exhibited varying degrees of structural disorganisation (Fig 4).

For each signal, discrete wavelet transform (DWT) was performed using the Daubechies order 6 orthonormal wavelet (D6). A D6 index was chosen as the wavelet represents the polynomial behaviour of the electrogram signal, exhibiting constant, linear and quadratic signal components.

This generated an 8 level decomposition of the original signal. Each approximation level represented a particular detail of the electrogram, such that a decomposition level of 1 (d_1_) could resolve fine details at the microvolt level, whilst level 8 (d_8_) was designed to resolve the coarse details. By summation of all decompositions derived from any one signal, a morphologically identical signal (a) to the original unfiltered signal (s) can be approximated.

### Frequency spectrum analysis (ii)—Fast Fourier Transform (FFT)

**Fig 5:**
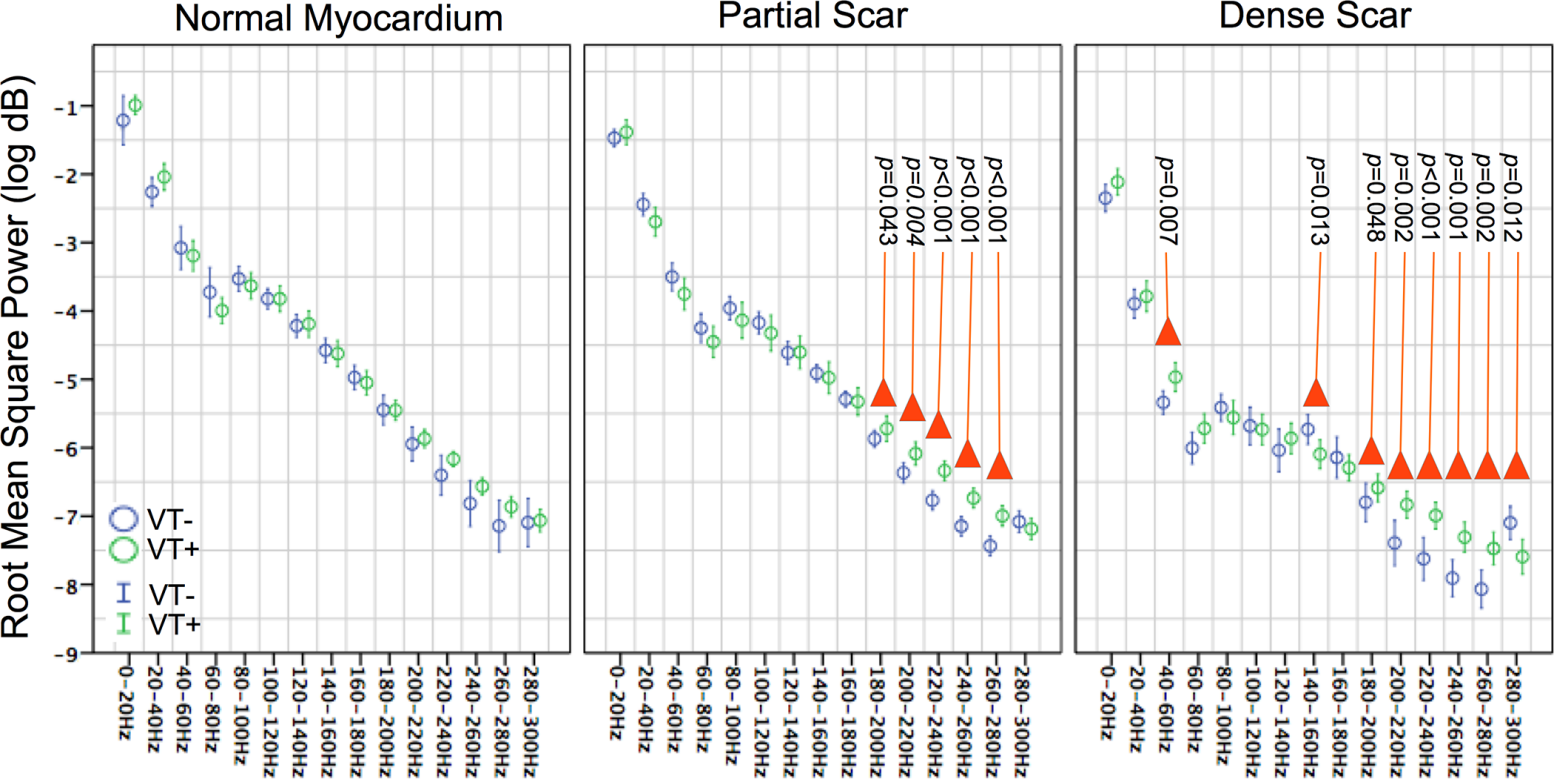
Group comparison of spectral power derived from electrograms recorded from within normal partial scarred, and densely scarred myocardium. *p*-Value < 0.05 shown, where significant differences in root mean square power (log dB) was observed between groups for each frequency band. Increased histological scar corresponded to greater differences in power observed between groups between 40-60Hz, 140-160Hz and 180-300Hz (mean values and 95% confidence intervals shown).

FFT was used to determine the frequency power spectra at each decomposition level of the DWT spanning a continuous 8±2 second epoch. The frequency spectrums of each composition level were divided into non-overlapping 20 Hz bands over the range of 0-300Hz and the root mean square (RMS) power in log dB was calculated for each band (Fig 5).

### Statistical analysis

For each sheep, the data consisted of transmural multi-electrode readings from 20 needles during sinus rhythm. The parameters: PNV, dV/dt_min_, and RMS power for each frequency band, were calculated from each electrode signal. The data was stratified based on histological scar percentage (normal = 0 to 10%; partial = >10 to 50%; dense = >50% scar). In addition, the histological heterogeneity (H_d_) for viable myocardium was assessed at each needle site.

**Fig 6:**
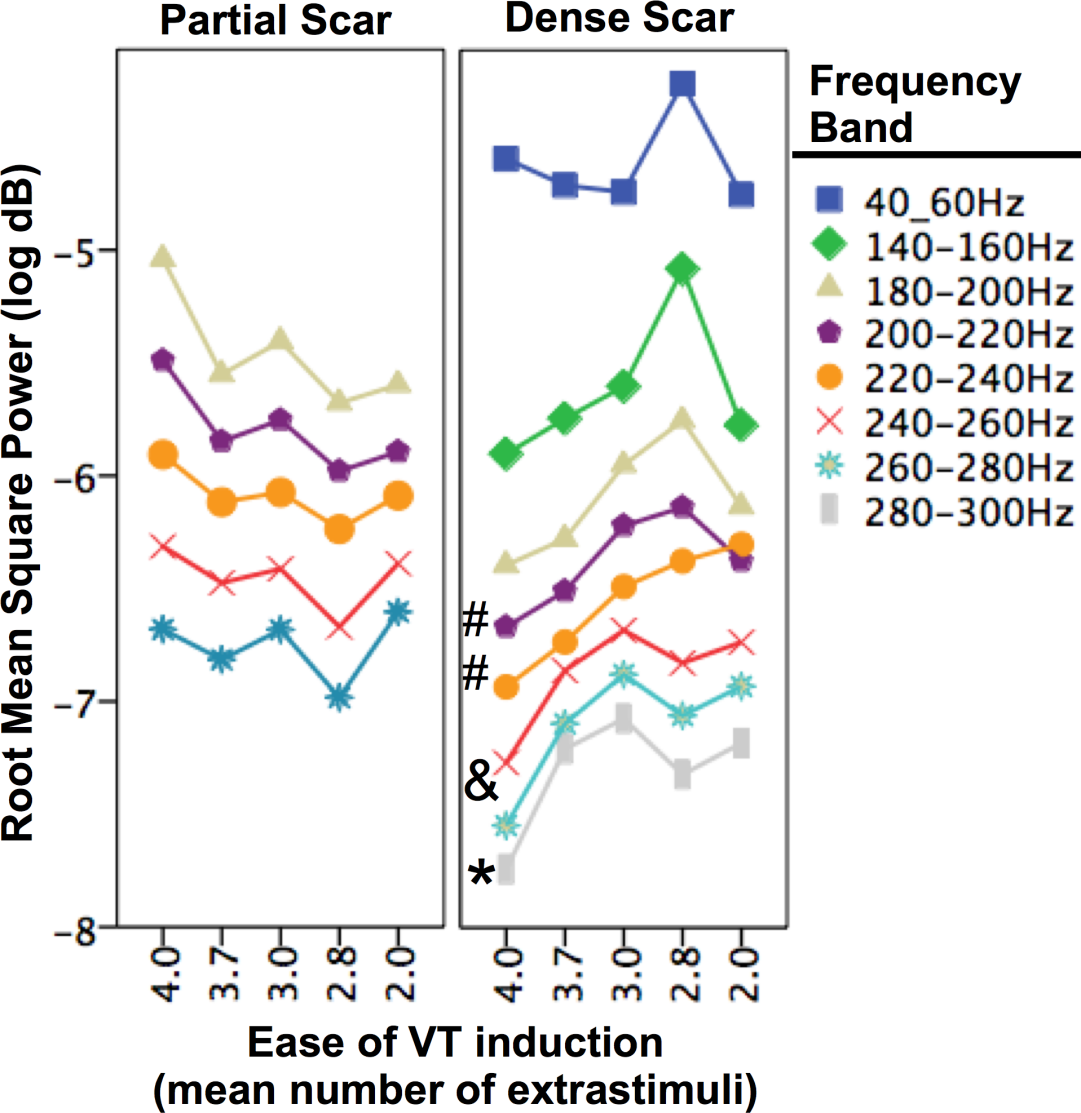
Relationship between the average number of extrastimuli required to induce VT and spectral power derived from electrograms recorded from within partially scared, and densely scared myocardium. Frequency bands which displayed significant differences between VT inducible and non-inducible groups are shown (refer to Fig 4). Symbols indicate probabilities of significant linear associations within specific frequency bands regarding ease of VT inducibility and spectral frequency attenuation, where # = *p*<0.001; **&** *p*<0.01; and * = *p<*0.05.

Statistical analysis was performed using the statistical package SPSS 22 (SPSS Inc. Chicago, IL). Kruskall-Wallis tests was used to compare the visual electrogram and spectral frequency characteristics between VT+ and VT- groups from within each histological stratum (Figs 2 and 5), and to assess the relationship between spectral frequency and ease of VT induction (Fig 6).

Curves for sensitivity and specificity were constructed using receiver operator characteristic (ROC) analyses to compare the predictive capability (area under the curve [AUC]) of electrophysiological criteria (including frequency bands which differed significantly between groups) for assessing the degree of structural heterogeneity (*H*_*d*_) of myocardium, and arrhythmogenic propensity of myocardial scar tissue (*Table 1)*. Supplementary analysis to determine the association between electrophysiological, and structural criteria was investigated using Spearman’s rank correlation coefficient (r). For Table 1, rank correlations were calculated individually within each sheep and summarized as the mean correlation coefficient. One-sample T-tests were used to test for significant departure from zero of the within-sheep rank correlations. For illustration (Fig 4), correlation coefficients were standardized (z-scores) prior to calculation of probabilities (p-value) for testing significance between groups (VT+/-) and time-points. A p-value <0.05 was considered significant.

**Table 1:** 

	*H*_*d*_				Arrhythmogenic Scar	
Criteria	*r ±* sd	*p*	AUC (95% CI)	*p*	AUC (95% CI)	*p*
**PNV**	0.56***±***0.19	<0.001	0.85 (0.82–0.88)	<0.001	0.51 (0.41–0.62)	0.823
**dV/dt**_**min**_	0.67***±***0.13	<0.001	0.90 (0.87–0.92)	<0.001	0.56 (0.45–0.66)	0.307
**RMS 40-60Hz**	-0.69***±***0.14	<0.001	0.89 (0.86–0.91)	<0.001	0.65 (0.55–0.76)	0.005
**RMS 140-160Hz**	-0.52***±***0.15	0.001	0.82 (0.78–0.85)	<0.001	0.69 (0.59–0.79)	<0.001
**RMS 180-200Hz**	-0.51***±***0.14	0.001	0.80 (0.77–0.84)	<0.001	0.62 (0.52–0.73)	0.026
**RMS 200-220Hz**	-0.50***±***0.14	<0.001	0.78 (0.75–0.82)	<0.001	0.72 (0.62–0.81)	<0.001
**RMS 220-240Hz**	-0.46***±***0.14	0.001	0.76 (0.72–0.80)	<0.001	0.77 (0.68–0.86)	<0.001
**RMS 240-260Hz**	-0.46***±***0.14	0.001	0.74 (0.70–0.78)	<0.001	0.72 (0.63–0.80)	<0.001
**RMS 260-280Hz**	-0.37***±***0.13	0.005	0.71 (0.67–0.75)	<0.001	0.71 (0.62–0.80)	<0.001
**RMS 280-300Hz**	-0.05***±***0.32	0.866	0.58 (0.53 0.62)	0.002	0.70 (0.60–0.79)	<0.001

Diagnostic performance of electrophysiological parameters for (1) discrimination of heterogeneous myocardial structure (*H*_*d*_) spanning the left ventricle and (2) discrimination of arrhythmogenic status (VT+/VT-) within the dense scar zone. *r* = mean spearman’s rank correlation coefficient. AUC = receiver operator characteristic curve Area Under the Curve. CI = confidence interval. Corresponding ROC AUC values for discrimination of Viable *H*_*d*_ are calculated based on a *H*_*d*_ cut-off value of 0.71 (based on the mean value across all specimens; range 0.34–2.9). Corresponding ROC AUC values for discrimination of Arrhythmogenic Scar are calculated based on arrhythmogenic status at EPS (VT+/VT-) as the descriminating index.

## Results

### Programmed ventricular stimulation

Monomorphic ventricular tachycardia that was sustained for >10 seconds was inducible in 7/14 animals post-MI. Induction of monomorphic sustained VT was reproducible in all 7 animals and reproducibly not inducible in the other 7 animals across the initial electrophysiology study and subsequent mapping study time-points. In the VT+ group, a total of 11 VT morphologies were inducible during the electrophysiology study whereas 18 morphologies were inducible during the mapping procedure. However, similar VT cycle lengths (electrophysiology study 222***±***45ms; mapping 259***±***43ms, p = 0.066) requiring the same number of extrastimuli (electrophysiology study 3.2***±***0.4; mapping 3.2***±***0.8, p = 0.984), were induced between follow-ups. Furthermore, the minimum S2-S4 coupling intervals used for successful induction of VT did not differ between these follow-up periods (electrophysiology study 175***±***33ms; mapping 186***±***27ms, p = 0.364).

### Histological assessment

In all animals, histological analysis confirmed the presence of scar in the anterior apical LV, consistent with an anterior MI following mid-LAD occlusion. A total of 282 tissue specimens that had corresponding reliable electrograms were analysed. The automated histological analysis technique was able to quantify the proportion of scar and degree of structural discontinuity (*H*_*d*_) on the histological specimen (Fig 1). There was no significant difference in collagen density relative to total scar (comprised of collagen, adipose tissue, and perimysium) area between VT+ and VT- groups (Fig 4A). Examining of the structural organisation of individual myocardial specimens revealed variation in *H*_*d*_ in both collagen dense areas, and sites situated remote from the infarct region (Fig 1). Overall however, relative to scar density, the VT+ group exhibited a significant proportional increase in *H*_*d*_ compared to the VT- group (Fig 4B).

### Visual electrogram characteristics

There was no significant difference in sinus cycle length between groups (VT+ group 703***±***72ms; VT- group 741***±***87ms; *p* = 0.385). As expected, unipolar PNV and dV/dt_min_ decreased inversely to scar percentage (Normal Myocardium vs Partial Scar PNV *p*<0.001, dV/dt_min_ p = 0.013; Normal Myocardium vs Dense Scar PNV *p*<0.001, dV/dt_min_<0.001) on the histological specimen (Fig 2A and 2B). However, no significant difference in these electrogram characteristics was observed between the VT+ and VT- groups (PNV *p* = 0.377; dV/dt_min_ *p* = 0.430).

### Frequency spectrum analysis

Differences in the electrogram frequency spectrum observed between VT+ and VT- groups are exemplified in illustration (Fig 5). Significantly greater RMS values were observed in the VT+ group as compared to the VT- group (see Fig 5 for p values). While this trend was observed across all tissue strata, significant differences were observed only within the infarct zone spanning certain frequencies within the range of 180-280Hz for partial scar, and 40-300Hz for dense scar. Greatest attenuation of spectral power was observed in the dense scar of VT- sheep.

To assess reliability of our methods, the frequency spectrum of signal elements identified using wavelet transform decomposition of the original signal into levels d_1_ –d_8_ was superimposable over the frequency spectrum of the actual complete signal (Fig 3). This indicates that no spectral leakage occurred during wavelet transform processing, and that the decomposed signal components encompassed spectral peaks, equal in power to the actual signal frequency spectrum.

**Fig 7:**
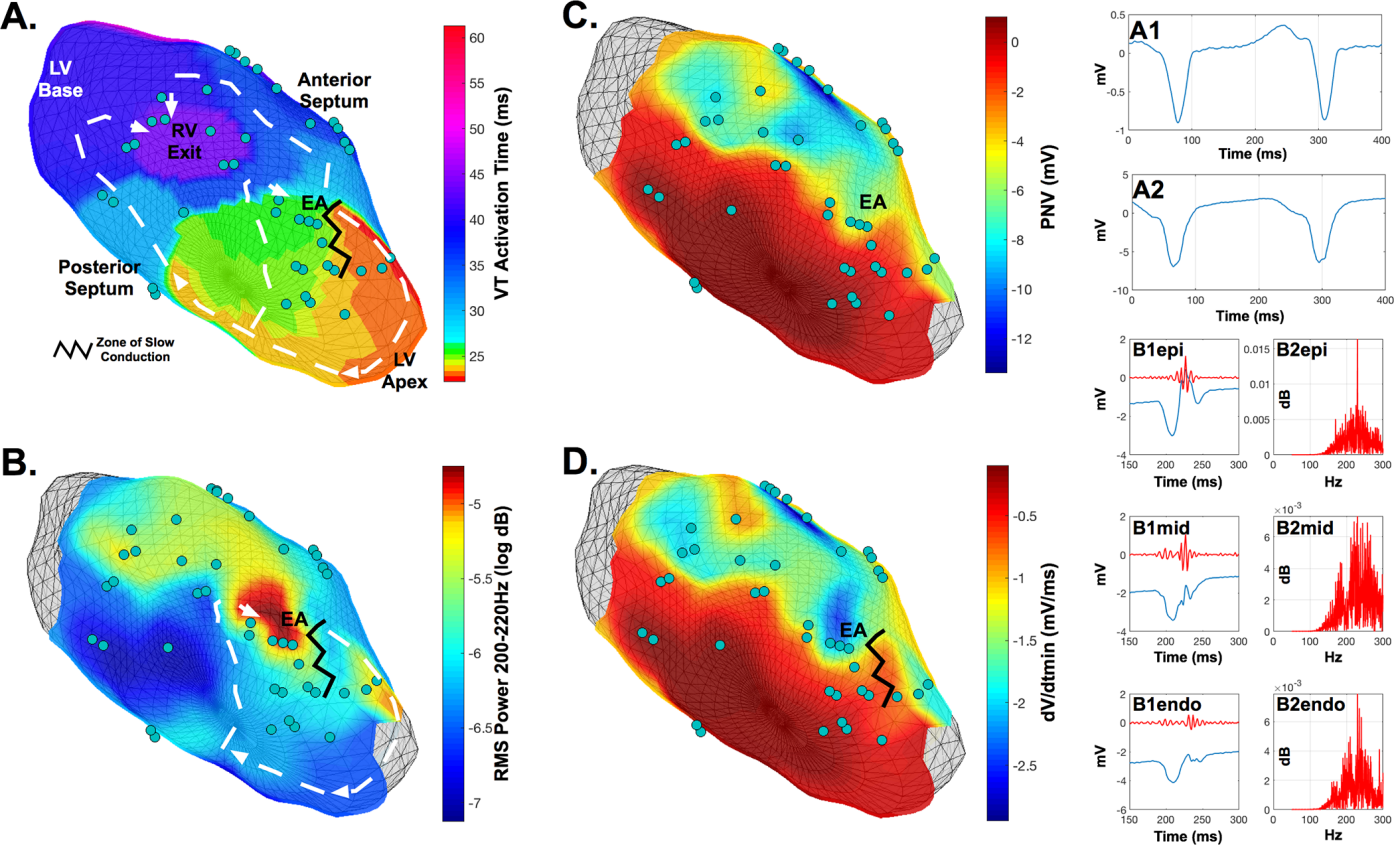
Example of left ventricular three-dimensional co-registration of VT re-entrant activation, electrogram frequency composition, and putative scar substrate. **A**. VT figure-of-eight re-entrant activation demonstrating earliest pre-systolic activation (EA) occurring at the antero-apical-septum. **A1**. Lead II surface ECG during VT. **A2**. Intracardiac unipolar electrogram during VT recorded from site EA. **B**. Observation of high spectral power density (RMS) in proximity to site EA spanning 220-240Hz of the local unipolar electrograms during sinus rhythm. **B1 panels**. Epicardial, mid-myocardial and endocardial unipolar electrograms recorded during sinus rhythm from site EA demonstrating delayed fractionated components and their associated d2 scale wavelet decomposition (red). B2 panels. Histogram of spectral frequencies of d2 scale decompositions demonstrating dominant frequencies occurring between 200 and 220Hz at the EA site. **C**. Corresponding voltage map indicating proximity of EA to the putative scar border zone. **D**. Corresponding dV/dt_min_ map demonstrating a high degree of electrical heterogeneity within proximity to EA. ● = plunge needle electrode recording sites.

**Fig 8:**
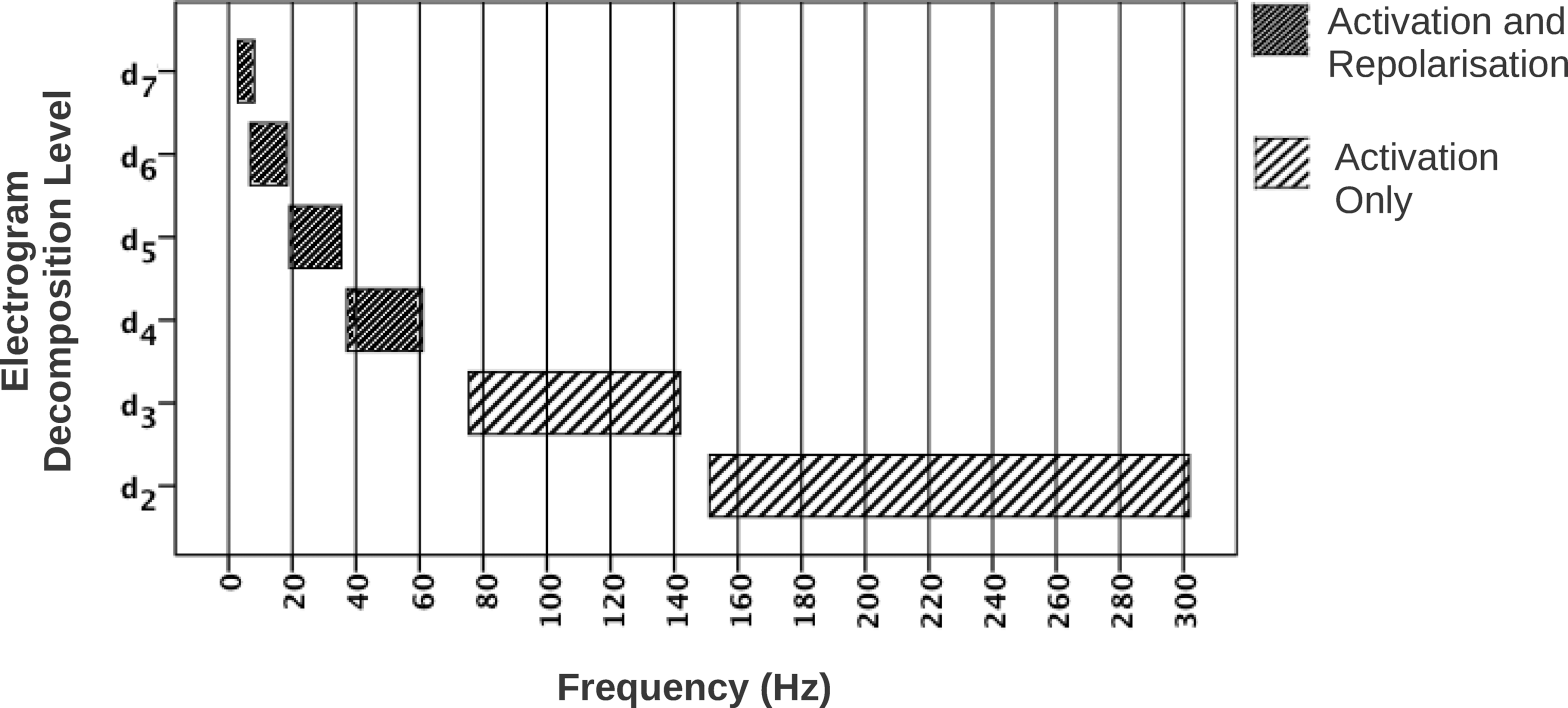
Range of dominant frequencies observed in all sheep at decomposition levels d_2_ –d_8_. Electrogram features extracted at levels d_1_ –d_3_ correspond to QRS components associated with activation only. Electrogram features extracted at levels d_4_ –d_8_ correspond to electrogram features spanning QRS and T wave segments.

Differences observed between groups for corresponding frequencies were temporally related to the QRS complex and activation (Fig 7*)*. By applying the wavelet analysis to our data set, we found that the frequency changes associated with the development of VT and scarring (~180-280Hz) were encompassed by decomposition level d_2_ (180-305Hz). Wavelet decomposition levels d_1_-d_3_ contain frequencies that were specifically associated with QRS morphology (Figs 3 and 8*)*. Lower frequency components associated with levels above d_3_ were of higher amplitude (mV) and representative of broader temporal aspects of the cardiac cycle inclusive of repolarization, but did not differ between groups.

### Assessment of infarct maturity on structural and electrophysiological characteristics

The time span between myocardial infarct induction and mapping did not have a significant impact on collagen density or *H*_*d*_ relative to scar area (Fig 4C and 4D). Similarly, the critical frequency ranges spanning 200-280Hz, associated with significant differences in spectral power between groups was not effected by infarct maturity (for all frequency bands *p* normal myocardium > 0.3; partial scar > 0.2; dense scar all > 0.3).

### Effect of myocardial structural organization on the electrophysiological frequency spectrum

Data regarding the effect of myocardial structural organisation on the electrophysiological properties are summarised in Table 1. As such, significant but moderate correlations were observed between the viable heterogeneity index (*H*_*d*_) and electrophysiological criteria including PNV, dV/dt_min,_, and electrogram spectral power spanning frequency bands that were observed to differ between groups with the exclusion of the 280-300Hz frequency band. Based on receiver operator curve analyses, all electrophysiological criteria, with the exclusion of spectral power spanning 280-300Hz, were reliable (all AUC > 0.71, *p*<0.005) at identifying myocardium that had a heterogeneity index (*H*_*d*_) > 0.71. Comparatively, the range of *H*_*d*_ indexes spanning all cardiac sites (infarct and remote-infarct) in all animals was 0.34–2.9.

Electrogram components within the spectral 40-60Hz range had the greatest correlation with *H*_*d*_. This effect was not specific to arrhythmogenic scar. In addition, reliable discrimination of arrhythmogenic scar from non-arrhythmogenic scar (i.e. compared between VT inducible and non-inducible groups) was not achieved using traditional discriminators of scar (PNV, dV/dt_min_). However, when confined to mapping within scar, electrogram spectral power spanning all frequencies (Table 1) were significant discriminators of arrhythmogenic scar, whereby the most reliable (AUC > 0.7, *p*<0.05) spectral components were in the high frequency range spanning 200-300Hz.

### Relationship between VT induction and the electrophysiological frequency spectrum

An inverse correlation between the number of extrastimuli required to induce VT and spectral power (RMS) was observed (Fig 6) within frequency spectrum bands that were influenced by VT propensity (Fig 5). This response which was predominantly observed across the 200-300Hz spectrum within densely scarred myocardium (Fig 6), was also confirmed to overlap the frequency spectrum that was associated with arrhythmogenic propensity (Fig 5), and sensitive to increased structural heterogeneity (*H*_*d*_) (Table 1). Furthermore, within this broad spectral range, the narrower spectrum of 220-240Hz demonstrated the greatest predictive power (AUC = 0.77, *p*<0.001) for discriminating between arrhythmogenic and non-arrhythmogenic scar tissue (Table 1).

The spatial assessment of the time-domain (wavelet decomposition features) and time-frequency components (frequency power spectrum) of unipolar electrograms recorded in sinus rhythm, relative to VT re-entrant propagation and putative scar, based on voltage mapping is exemplified in illustration (Fig 7). Consistent with observations from all animals, regions with electrograms demonstrating increased spectral power between 220-240Hz were often observed at sites that activated within the earliest pre-systolic phase of the VT cycle. Such sites demonstrated complex and delayed fractional components within the local electrograms, were within proximity to zones of slow conduction, and adjacent to spatially heterogeneous regions of scar.

## Discussion

This *in vivo* study employed direct correlation of histology with spatio-multiresolution analysis of unipolar electrograms in a post-infarct, large animal model to examine the underlying mechanistic relationship between fractionated electrogram components, structural organisation of the myocardium and the ability to induce VT.

The main findings of this study were threefold, (1) visual characteristics of unipolar electrograms were quantitatively influenced by the degree of myocardial heterogeneity yet were unreliable discriminators of arrhythmogenic capacity of the tissue. (2) The spectral frequency characteristics within the high-frequency bandwidth of unipolar electrograms were sensitive to changes in myocardial heterogeneity, although in contrast to visual electrogram characteristics, had greater discriminatory capability in identifying arrhythmogenic substrate. (3) High frequency spectral components of unipolar electrograms recorded from within dense areas of scar correlated in magnitude with the degree of effort (number of extrastimuli) required to induce VT during the electrophysiology studies.

Changes to the electrogram have been described in association with disruption of the wavefront passing through fibrotic changes in the micro-architecture of the myocardium. [2, 7, 10, 14, 26] Newer models have applied sophisticated signal analysis techniques but have not been validated against histological findings in an *in vivo* model of post-myocardial infarction.[17, 18, 27] To the best of our knowledge, our study demonstrated a novel quantitative approach for assessing the architectural organisation of the myocardium.

In order to define electrogram characteristics that are suggestive of fractionation, we undertook our analysis using similar analytical techniques which were sensitive at uncovering subtle changes in the electrogram. Furthermore, we analysed these data with respect to the histological properties measured in detail at each electrode site. The methods employed for automated quantitative analysis of histology and electrograms were devised to minimise operator bias.

Discrete wavelet transform was performed to extract multi-level morphological features from electrograms recorded for all intramural layers of the left ventricle. FFT was then applied to assess differences in electrogram spectral characteristics at each level of detail extracted during DWT, and to determine whether these features were attributed by the effects of myocardial structural organisation and arrhythmogenic potential. From this, the RMS power of the frequency spectrum may be interpreted as a surrogate marker of wavefront complexity.

Fractionated electrograms have been found to be caused by electrode movement relative to the movement of the myocardium.[27, 28] By employing transmural plunge needle mapping in our experiment, such signal artefact was avoided as a result of stable electro-myocardial interfacing. Furthermore, needle mapping co-registration facilitated direct correlation of electrophysiological and local histological information.

### The effect of myocardial structure on electrogram morphology

As expected, attenuation of electrogram amplitude (PNV) and broadening of the intrinsic deflection (dV/dt_min_) were associated with increased scar density, and structural heterogeneity, yet were not associated with arrhythmogenic status. Given that the relationships between scar density and electrogram amplitude or dV/dt_min_ were similar between groups, we postulate that electrophysiological pertubations associated with arrhythmogenicity are more complex than that attributed to the presence of collagen or degree of myocardial heterogeneity alone.

Thus, in agreement with Cabo *et al*., we have shown that dV/dt_min_ or PNV is not an optimal classifier of local or distant activity in scar tissue.[29] In agreement with our hypothesis, the authors concluded that the frequency component of unipolar signals obtained using fast fourier transformation provided better discriminatory capability than dV/dt_min_ whereby high spectral frequencies were more frequently observed within proximity to the source of fractionation.[29]

### Spectral characteristics of arrhythmogenic myocardium

When comparing the VT inducible and non-inducible groups, significant differences in RMS power in the higher frequencies were noted in areas of scar. When the electrogram was deconstructed with DWT analysis, the frequency range where these differences were observed corresponded to microvolt and millivolt features within the QRS complex. Given that, relative to the amount of scar, the degree of heterogeneity and magnitude of fractional electrogram components (high frequency) increased in animals with inducible VT, we hypothesise that the observed increase in activity (fractionation) within the high frequency spectrum is representative of enhanced discontinuous local propagation, a key factor in the formation of re-entrant VT. In this study, we provided further evidence of this mechanism by demonstrating that increased fractionation was also associated with ease of VT induction.

Our findings are supported by theoretical models that identify similar high frequency perturbations in the unipolar electrogram. In a study by Kapela *et al* employing a Luo-Rudy dynamic cell model simulating unstable wavefronts, small timing fluctuations were represented by discontinuous propagation resulting in local high frequency (100-300Hz) activity.[18]

Frequencies similar to those observed at the cellular level *in silico* have also been reported by other researchers using high resolution ECGs. A study by Popescu *et al* was able to differentiate between arrythmogenic and non-arrhythmogenic subjects by identification of high frequency changes (200-300Hz) using high resolution ECG.[30] Similar to our study, they employed multi-resolution analysis using wavelet transform to translate characteristics of the frequency spectrum into corresponding temporal features of the ECG.

Whilst the spectral characteristics measured in our study were consistent with the abovementioned studies by Kapela and Popescu *er al*., we observed greatest sensitivity and specificity over a narrower bandwidth spanning 220-240Hz. We attribute this to potentially more sensitive and specific electrogram measurements inherent to intracardiac plunge needle electrodes compared to signals recorded on the skin surface.

Each of the abovementioned studies by Kapela, Popescu, and Tsutsumi, have applied the classical Morlet wavelet-based technique for uncovering high frequency sources from electrograms. In this study, we applied the Daubechies wavelet transform due to the superior temporal resolution compared to the Morlet wavelet transform. Senhadji *et al*., compared the ability of Daubechies, spline and Morlet wavelet transforms to recognise and describe features in isolated cardiac beats in ischaemic and non-ischaemic patients.[31] Their study concluded that Daubechies transform had superior discriminatory capability compared to the spline and Morlet wavelet transforms.

Electrogram fractionation measured using bipolar and unipolar mapping modalities has been previously confirmed to be associated with histological scarring post-MI.[14] We extend this knowledge by systematically comparing the degree of fractionation observed in VT inducible and non-inducible animals, and demonstrated that increased propensity for arrhythmogenesis is correlated with increased fractionation.

More recently, Campos and colleagues compared both time-domain (based on number of signal deflections) and frequency-domain (dominant frequency assessment by FFT) methods of quantifying bipolar electrogram fragmentation to determine the relationship of fragmented potentials to the VT isthmus in patients with post-infarct cardiomyopathic VT.[11] In that study, it was concluded that time-domain methods were superior than frequency domain methods in uncovering critical VT isthmuses due to the introduction of harmonics in the frequency domain that may be generated by the presence of high amplitude morphological features. In our study, we overcame three key limitations reported by Campos *et al*., 1) by utilising unipolar electrograms we were able to negate the influence of the directional nature wavefronts which may impact on the amplitude and subsequent accuracy of bipolar recordings; 2) by performing DWT to decompose the electrogram into constituent multi-resolution components prior to performing FFT, we were able to eliminate spectral leakage and harmonics that may have been generated by low frequency or high amplitude morphological features, and which may have impacted on the frequency composition identified for high frequency and low amplitude morphological features; and 3) by comparing signal compositions between animals with inducible VT and an appropriate control group of animals without inducible VT we were able to quantitatively demonstrate the subtle differences in electrogram frequency-domain characteristics between these two groups of animals with respect to myocardial structural properties.

The abovementioned studies have implicated fibrosis and scarring as the major factor towards fractionation however, our findings confirm that, in addition to the composition, the structural organisation of myocardium, as determined by the heterogeneity index is an important contributing factor to fractionation and ventricular tachycardia. Furthermore, we demonstrated that between VT inducible and non-inducible animals, the dense scar zone had the greatest disparity in high frequency spectral components relative to other ventricular sites assessed, suggesting that microreentrant circuits or channels, may be highly prevalent within this region of scar.

The evidence of conducting channels within densely scarred myocardium and their potential as targets for therapeutic application is supported by clinical studies, which have demonstrated the potential benefits in de-channelling of such circuits by radiofrequency ablation, for reducing VT recurrence.[32] Indeed, one of the potential challenges of targeting arrhythmogenic circuits within densely scarred myocardium using current clinical approaches involving mapping of late abnormal potentials, is that subtle but discrete potentials may be masked out visually by the increased QRS width observed at such sites.[32] As such, our method of spectral frequency based unipolar mapping using catheters with encompassing small electrodes with surface areas of approximately 3.8mm^2^ may be one approach which may be utilised to unmask the non-visual or hidden components associated with arrhythmogenic substrate.

### Multifactorial mechanism of fractionation

Non-uniform slowing of conduction, such as that caused by structural inhomogeneity and myofibre branching, as described from cellular preparations is the primary contributing factor to the fractionation that we observed *in vivo*.[33]

The subtle, differences between groups in non-infarcted tissue that were similar in pattern to that observed within scarred regions, although not significant, suggest that there may be other non-injury or compensatory related mechanisms involved.[34, 35] Based on diffusion tensor imaging in the ovine model, previous studies have demonstrated that significant transmural reorganisation of the ventricular myofibre architecuture occurs remote from the scar zone in post-infarct remodelling.[36] This, in turn may be associated with reactive interstitial fibrosis that can occur remote from the infarct region and may include interstitial sites such as the perimysium.[37] However we were unable to demonstrate corresponding effects on the power spectrum due to the relative increased electrogram amplitudes remote from infarcted tissue, which oversaturated the low-amplitude high-frequency components, thus attributing to increased variability in power across the frequency spectrum.

In contrast to Bogun *et al* we found that there was no significant increase in heterogeneiety that could contribute to increased fractionation with respect to infarct age.[10] Our studies differed in electrogram collection where we gathered Electrograms from unipolar plunge electrodes, thus ensuring a stable myocardial to electrode interface, whereas Bogun *et al*, employed bipolar endocardial mapping to obtain their data which in turn can compromise signal quality during myocardial wall motion related catheter skating and varying orientation of the bipoles with respect to the angle of the activation wavefront. Despite the differences in recording modalities used, the absence of temporal effects observed in our study could be due to the relatively short follow up period in our animal study as compared to their clinical observations. In addition, this may also account for the non-significant effect of infarct maturity (during the short follow-up period) on the other structural and electrophysiological properties investigated in our study. This finding supports the hypothesis that VT circuits, once established, remain persistent due to the relative temporal stability of the substrate within the first few months after myocardial infarction.[38]

### Clinical implications

A critical limitation in identifying, and thus selecting, those at risk of suffering from post-infarct sudden cardiac death is an understanding of the mechanism that leads to re-entrant ventricular arrhythmias. The current study identified unipolar frequency characteristics that differ in those hearts capable of maintaining sustained monomorphic VT versus those that cannot. These differences could not be explained by scar macro-architecture (scar content) as they were also present in areas of normal healthy myocardium, suggesting a possible molecular or microstructural mechanism such as anisotropic remodelling involving remote infarct regions of ventricle.

It is worthwhile noting that many Electrophysiological laboratories today routinely filter out the high frequency spectrum to minimise noise and artefact. However, such rigorous filtering may eliminate an important source of data. Hence, the applicability of utilising FFT and DWT may be made possible using less aggressive low-pass filtering setting; this would allow high frequency distributions to be analysed in predicting VT inducibility.

Although this method has only been tested using plunge neede electrodes, such signal analysis methods may be directly applied to catheters employing tip-deployable plunge needles as previously described by AbdelWahab *et al*., for use in selecting transmural ablation targets for ventricular tachycardia.[39]

### Limitations

It is likely that smaller band sizes may have uncovered greater differences in the electrogram spectrum between groups. To date there is no systematic method of determining the correct band size and others seem to adjust the band size depending on the EGM properties that are under observation. In our situation, larger band sizes were applied to unmask significant trends rather than erroneous deviations.

Given that fractional electrogram morphology was not confined to areas of fibrosis in the VT inducible group, it is hence likely that left ventricular remodelling in our ovine model, may involve widespread molecular and/or cellular modifications beyond the scope of this study.

In this investigation, the range of histological sampling periods were variable. It is well established that dramatic changes in collagen content (and cross-linking) occurs as the scar transitions from the proliferative to the maturation phase of development. Despite the variation in sampling time-points within each group, there was no statistical difference in procedural timing between groups in regards to EPS and mapping studies. All procedures were timed beyond the initial inflammatory phase. Interim EPS procedures were timed within the window of transition from the proliferative to maturation phase, and mapping procedures involving histological sampling were all performed during the maturation phase of remodelling as supported by similarities in tissue heterogeneity (*H*_*d*_) observed with respect to scar area and infarct maturity (Fig 4). In addition to this, we did not observe any significant interstitial scarring remote from the infarct zone, possibly due to the juvenile age (1–4 years) of the animals at the time of infarction, and thus may not necessarily reflect infarct pathophysiology associated with clinical myocardial infarction within a mature age population.

It should also be addressed that the plunge needle electrodes could have possibly injured the myocardium and thus may have affected the results, however we have previously demonstrated that plunge needle mapping is relatively non-detrimental to cardiac conduction *in vivo*.[40] Another potential drawback of these needle electrodes is that they are not used for clinical mapping. However, in an experimental setting, one of the great benefits of these electrodes is the ability to retrieve direct histological specimens from the area surrounding the needle.

## Conclusion

Combined DWT and FFT analysis has allowed us to closely scrutinize Electrograms to the microvolt level. Coupled with correlation of histological data, this provides a powerful tool to understand and postulate the underlying mechanisms involved in arrhythmogenesis. We have identified changes to the frequency spectrum that herald differences in myocardium that will develop VT as compared with myocardium that will not. These changes in the high frequency domain are likely to be of multi-factorial aetiology. Whilst further research efforts will be required to elucidate the exact pathogenesis, we believe that it represents a complex interaction between many factors that have been linked with ventricular arrhythmias. The zig-zag propagation through anisotropic tissue as well as electrotonic modulation are all likely to be implicated in this highly complex process.
